# Test Generation Algorithm for Fault Detection of Analog Circuits Based on Extreme Learning Machine

**DOI:** 10.1155/2014/740838

**Published:** 2014-12-29

**Authors:** Jingyu Zhou, Shulin Tian, Chenglin Yang, Xuelong Ren

**Affiliations:** School of Automation Engineering, University of Electronic Science and Technology of China, Chengdu 611731, China

## Abstract

This paper proposes a novel test generation algorithm based on extreme learning machine (ELM), and such algorithm is cost-effective and low-risk for analog device under test (DUT). This method uses test patterns derived from the test generation algorithm to stimulate DUT, and then samples output responses of the DUT for fault classification and detection. The novel ELM-based test generation algorithm proposed in this paper contains mainly three aspects of innovation. Firstly, this algorithm saves time efficiently by classifying response space with ELM. Secondly, this algorithm can avoid reduced test precision efficiently in case of reduction of the number of impulse-response samples. Thirdly, a new process of test signal generator and a test structure in test generation algorithm are presented, and both of them are very simple. Finally, the abovementioned improvement and functioning are confirmed in experiments.

## 1. Introduction

For testing of integrated circuits (ICs) and system on chips (SoCs), both test accuracy and costs are important. On the one hand, it is not cost-effective to classify analog circuits subject to parametric faults if test application takes a long time along with increasingly rapid application of ICs and SoCs. On the other hand, it is difficult to apply traditional functional testing to ICs mixed with SOCs, as functions of which are too complex to test. Thus, this test generation algorithm is proposed as a new test strategy [1–5]. Previously, this method is used to classify faults based on signal response by inputting different sine waves. Balivada et al. propose using some identified signals to obtain indexes such as delay and rise time, in order to calculate different parameters for signal classification in 1996 [3]. Hamida et al. put forward and developed methodology and software for sensitive testing for ICs in 1996 [4]. However, it hardly becomes automatic due to limitations. In 1995, Devarayanadurg and Soma put forward a methodology of minimum/maximum testing for ICs which functioned well [5] and required establishment of a response space of device under test (DUT) for simulation. However, such requirement is of too high time cost for large circuits. There are a countless number of these kinds of methods. Analog test generation differs from classical digital test generation. This classical digital test generation shall only consider fault models such as 0-1 faults, delay faults, and bridge faults. Test generation algorithms for analog circuits are always more difficult than classical digital test generation algorithms, due to a lot of problems such as continuous-time waveforms and tolerance for parameters.

Analog test technology mainly includes two parts: test generation and fault diagnosis. There are many researches on fault diagnosis [6–16]. Yang et al. propose a fault dictionary method about analog circuits in 2009 [7, 8]. This method is simple, but it is relatively complicated for establishment of this dictionary. In 2011, Ting proposes an analog circuit fault diagnosis method based on component connection model (CCM) [6], which can properly realize fault location. However, the scale of CCM will become larger with the increase of circuit complexity. Li et al. put forward a test method for the optimal noise estimation through Kalman filter in 2013 [9]. In this method, various noise interferences in the test are taken into full account, but the process of noise estimation is comparatively complex. Nowadays, to introduce machine learning into analog circuit test served as a hotspot of fault diagnosis researches, especially for wide application of support vector machine (SVM) as an effective classifier [10–16]. These methods are characterized with classification of various fault features through classifier for the purpose of fault diagnosis. This category of methods is always of favourable test effects. However, the research emphasis of these methods is centred on fault diagnosis. As a result, test generation is neglected.

Analog test generation algorithm is mainly to introduce the concept of digital test generation into analog circuit test. The difference lies on the said analog circuit fault diagnosis method. The main purpose of analog test generation algorithm is to generate test signals necessary for fault diagnosis. Test signals here must be able to motivate fault. In other words, fault should be made to be detectable, so as to structure a fast and convenient online test platform. In recent years, fewer researches are engaged in analog circuit test generation. Pan and Cheng propose a cost-effective method to detect parametric faults in linear time-invariant (LTI) analog circuits in 1999 [1]. This method differs from traditional techniques and uses a limited number of derived test patterns as input stimuli and unprocessed DUT outputs for fault detection and classification by using perceptron. This method has good classification performance, but inadequate testing accuracy. Long et al. propose a test generation algorithm for analog systems based on support vector machine (SVM) in 2011 [2]. This algorithm with high testing accuracy uses SVM to map the lower-dimensional nonlinear response space into the higher-dimensional feature space for effective classification. However, this algorithm has much higher time cost because of complex computation of SVM, processes of test signal generator and test structure, and has unstable testing accuracy because of reduced precision in case of compressing the sampled space.

In order to solve the abovementioned problems, a new test generation algorithm based on extreme learning machine (ELM) [17, 18] with much lower time cost and simpler processes has been proposed in this paper. Furthermore, trade-off parameters are not sensitive to the accuracy of classification [19, 20] due to the necessity of being specified by users, and so ELM has good classification performance without optimization of trade-off parameters in case of compressing sampled space.

The rest of this paper is organized as follows.  Section 2 is about overview of test generation algorithms.  Section 3 introduces the novel test generation method based on ELM.  Section 4 elaborates structure and analysis of this algorithm.  Section 5 describes the performance of the proposed method through simulation.  Section 6 includes conclusions.

## 2. Overview of the Test Generation Algorithm

Basic structure of the test generation algorithm is to transform analog circuit test into digital field for analysis through digital-analogy and analog-digital converter. Normal state and various fault states of circuits can be regarded as an isolated state circuit. Circuits in different isolated states are sampled, so as to obtain abundant impulse response vectors. Then, impulse response vectors in normal state and fault state are separately identified, so as to establish different impulse response spaces. At last, a classifier is used to divide different sampling spaces. Test signals are obtained through calculation from trained classification hyperplane expression, so as to stimulate tested circuits. A comparison is made between output voltage and threshold value through a comparer, so as to realize fault diagnosis.

**Figure 1:**
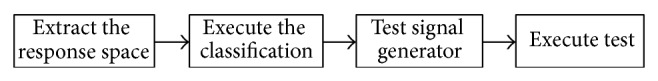
Test generation framework.

An overview of the test generation framework for LTI analog circuits is shown in Figure 1.

Core steps of the test generation algorithm include two aspects: the one is to work out various classification hyperplanes and to figure out proper test signals as input of tested circuits according to classification features of state of various circuits (normal state and fault state); the other one is to analyze output of tested circuits after being stimulated by test signals and then judge any fault of such circuits. As shown in Figure 1, the test generation method contains mainly four steps as follows.

**Step 1 (extract response space constructed by sampled impulse responses for classification).**

The first step is to build a response space constructed by many response vectors, which are constructed for each instance by sampling the analog circuit output signal. As the bandwidth (BW) is much smaller than the sampling frequency, there are a large number of impulse-response samples. In order to reduce costs, a new space of impulse-response is constructed by extracting the original space of sampled impulse-response [1]. A decreased number of samples may lead to loss of classification information. To solve this problem and keep classification accurate in the case of compressing, a lot of time shall be spent on optimization algorithms.

**Step 2 (execute classification).**

As test generation for LTI analog can be viewed as one of several two-class classification problems, accuracy is important for each classification method. The SVM-based test generation algorithm has good classification accuracy [2].

In test generation, two-class classification shall be executed for *N* times for the sake of calculating *N* hyperplanes in order to classify *N* circuit instances with parametric faults into normal circuit instances. Then, linear hyperplane is found by training sets, and test efficacy depends on classification precision through test sets. Therefore, for test generation, the whole time cost of classification is an important factor.

**Step 3 (generate test signals).**

Test signals are obtained from classification hyperplane. The nonlinear classification algorithm is adopted for test generation in order to improve classification accuracy [2], but this algorithm deals with classification problems by mapping the original space to a higher dimension of feature space; that is to say, it obtains a linear classification hyperplane in a higher dimension of space. Therefore, in order to generate test signals, we need to calculate the classification hyperplane in linear dimension from the linear classification hyperplane in a higher dimension of space, which is a very complex process.

**Step 4 (execute test).**

In order to determine whether circuit under test (CUT) passes or fails, we need an impulse as a threshold to compare test signals generated in Step 3 through CUT, analog-to-digital converter (ADC), and digital-to-analog converter (DAC). Therefore, *N* instances need *N* thresholds. If it is hard to compare by this threshold, test process would be more complex.

To solve the abovementioned problems that have been ignored in previous test generation methods, a new test generation algorithm based on ELM has been proposed in this paper.

## 3. Test Generation Algorithm Based on ELM

### 3.1. ELM Algorithm

The test generation algorithm proposed in this paper is based on the ELM, which is introduced as follows.

The ELM [17, 18] proposed by Huang et al. is a novel learning method for single-hidden layer feed-forward neural networks (SLFNs). According to the theory of ELM, in the case of activated activation function which is infinitely differentiable, if the number of samples is equal to that of hidden nodes, that is, $N=\tilde{N}$, matrix *H* is square and invertible and SLFNs can approximate these samples with zero error. However, the number of hidden nodes is much less than that of samples in most cases. According to Huang's theory, *H* is a nonsquare matrix, $\tilde{N}\ll N$, and the smallest norm least-squares solution is

(1) $$\begin{array}{c} \hat{\beta }=H^{+}T, \end{array}$$

where *H* ^+^ is the Moore-Penrose generalized inverse of matrix *H*. According to the above derivation, the output function of ELM is

(2) $$\begin{array}{c} f\left(x\right)=\overset{L}{\underset{i=1}{\sum }}\beta_{i}G\left(a_{i},b_{i},x\right)=\beta \cdot h\left(x\right), \end{array}$$

where *β* is the output weight vector between hidden layer and output layer and *h*(*x*) is an activation function. Here, *h*(*x*) actually maps the data from the *d*-dimensional input space to the *L*-dimensional hidden-layer feature space (ELM feature space). For binary classification application, the decision function of ELM is

(3) $$\begin{array}{c} f\left(x\right)=\mathrm{sign}\left(\overset{L}{\underset{i=1}{\sum }}\beta_{i}G\left(a_{i},b_{i},x\right))=\mathrm{sign}(\beta \cdot h\left(x\right)\right). \end{array}$$

According to the above theories, ELM is different from other algorithms based on SLFNs, such as back propagation (BP) algorithm which contains five limitations: (1) different learning algorithms for different SLFNs; (2) being time-consuming; (3) gradient-descent/iterative approaches; (4) over fitting; and (5) local minima.

For ELM, parameters of input layers are randomly chosen, while output weight vectors are obtained by calculating output matrix of the hidden layer, which is the Moore-Penrose generalized inverse of hidden layer output matrix. Therefore, only hidden nodes shall be assigned. Compared to SVM, ELM is advantageous from three aspects: (1) being less time-consuming, (2) having higher generalization performance, and (3) having milder constraint to parameters.

### 3.2. Extract Response Space

When classification accuracy is maintained, we need to build the new impulse-response space by sampling impulse responses for classification and then compress the original impulse response.

The main idea of [1] is about cost efficiency to save time and space. In this theory, the selected impulse sampled space is recompressed by selecting parts of elements equally from response impulse vector space *h* to form new sampled vector space. For example, (*h*[0], *h*[4], *h*[8], *h*[12],…) is a new sampled vector after extracting samples with a distance interval of 4, and these vectors can then be rewritten as (*h*0, *h*1, *h*2, *h*3,…). This method makes the accuracy of test performance uncertain although it can be applied easily. Therefore, it is important to keep high test accuracy under compressing.

**Figure 2:**
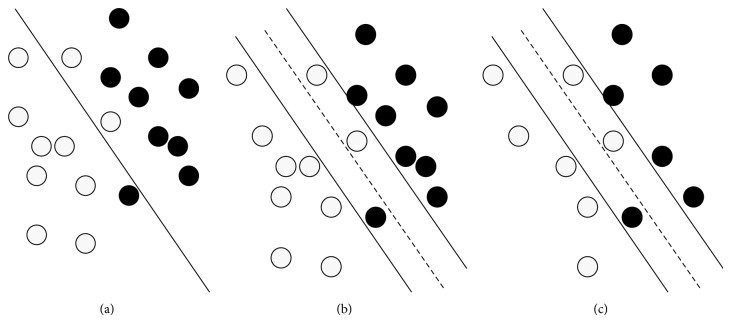
(a) Normal hyperplane classification; (b) hyperplane classification with supporting planes; (c) hyperplane classification with supporting planes in the case of compressing.

Considering that there is an ideally selected impulse sampled space, select parts of elements equally from it. As shown in Figure 2, comparison is made between mapping linear spaces after element selection and the original mapping linear space.

As shown in Figure 2(a), the lower-dimensional nonlinear response space is mapped into the higher-dimensional feature space for the sake of effective classification. Some dots may drop into new supporting planes in higher-dimensional linear feature space. According to Figure 2(b), trade-off parameters such as the trade-off factor *γ* and cost factor *C* in SVM are introduced for rightful classification, in order to evaluate introduction error of outliers experientially [19–22]. As shown in Figure 2(c), outliers on classification may have greater influence when samples of the feature space are compressed.

Based on the above analysis, in order to obtain better classification accuracy for different features, different trade-off parameters shall be introduced under relaxed conditions. For the sake of accuracy of the entire algorithm, contradiction between compressed samples and changed supporting planes can be eased more efficiently through experienced trade-off, by introducing different trade-off parameters under relaxed conditions.

Let us further discuss the changed trade-off parameters. The influence on trade-off parameters mainly comes from information loss because of compressing the sampled space. Therefore, methods can be applied to select sampled vectors with more information [23] and optimize parameters into trade-off ones [24]. However, these methods increase complexity of the algorithm.

According to Huang's theory, trade-off parameters are more sensitive to classification accuracy in SVM, lest squares support vector machine (LS-SVM), and ELM based on Gaussian kernel function, but are less sensitive to ELM based on both Sigmoid and multiquadric activation functions [19, 20]. Based on the above analysis, classification risk may be reduced by applying an algorithm with a lower sensitive relationship between testing generation accuracy and trade-off parameters under compressed sample space. Therefore, compared with other algorithms mentioned above, classification method of ELM which is based on both Sigmoid and multiquadric activation functions is a better choice in the case of compressing in order to maintain classification accuracy.

### 3.3. Execute Classification by ELM

#### 3.3.1. Time Cost

In the test generation, two-class classification shall be executed for *N* times for the sake of calculating *N* hyperplanes in order to classify *N* circuit instances with parametric faults to normal circuit instances. Then, each linear hyperplane is found by training sets, and test efficacy depends on classification precision through test sets. Therefore, for test generation, the whole time cost of classification is an important factor. In order to reduce time cost, the method of compressed sampled space is applied to test generation. However, time cost of classification methods has been ignored in previous algorithms. This paper applies a recently found fast-speed learning method called ELM to classification.

Time cost of classification algorithm is mainly caused by computational complexity. The number of impulse samples for classification is assumed to be *N*. Usual activation function *h*(*x*) can be used to calculate ELM directly. Therefore, computational complexity mainly comes from linear equations, the size of which is $\tilde{N}\times \tilde{N}\;(\tilde{N}\ll N)$ [19].

Computational cost of SVM mainly comes from calculation of the Lagrange multipliers *a*, while that of which for SVM mainly comes from the solution with convex quadratic programming to a matrix of sampled space with a size of *N* × *N* × 2 [21]. If *N* is large, the computational costs mainly come from solution to the matrix with a size of (2*N* + 1)×(2*N* + 1) × 2. Therefore, classification of SVM may have much greater computational complexity.

Therefore, in terms of computational complexity of learning algorithm, ELM-based classification algorithm is less time-consuming than SVM.

#### 3.3.2. Choice of Activation Function and Kernel Function of ELM for Proposed Algorithm

ELM includes two kinds of output functions, namely, the non-kernel based output function which maps *h*(*x*) and is known to users and kernel based output function which maps *h*(*x*) and is unknown to users. This paper puts forward an ELM-based algorithm for LTI analog circuits, and traditional activation functions such as Sigmoid and Gaussian functions can be easily used to map *h*(*x*). Furthermore, trade-off parameters of ELM, which has kernel based output functions, such as Gaussian kernel function, are more sensitive to classification accuracy under compressed sample space. So, the non-kernel based ELM (described in Section 3.1) is a better choice.

ELM is one of the best classification algorithms based on activation functions. Normal activation functions include Sigmoid function, hard-limit function, Gaussian function, and multiquadric function. According to analysis of compressing sample space, ELM based on both Sigmoid and multiquadric activation function is better than others. However, ELM based on Sigmoid activation function is better than that based on multiquadric activation function in terms of classification accuracy in circuit test. Therefore, the Sigmoid activation function suits ELM in circuit test.

In general, the non-kernel based ELM with Sigmoid activation function is suitable for algorithm proposed in this paper.

### 3.4. The Novel Method of Test Signal Generator Based on ELM

Considering input stimulation *x* and impulse response *h*[*m*] in the analog test generation algorithm, the output response of LTI can be expressed as follows:

(4) y[n]=∑m=0∞x[n−m]·h[m]≈∑m=0d−1xn−m·hm n=0,1,…,∞.

*h* = *h*[0], *h*[1],… stands for the sampling vector of a circuit impulse response. It can be known from the circuit theory that output response *y*[*n*] is mainly influenced by *h*[0], *h*[1],…, *h*[*d* − 1], in which *d* = *F*_*s*_/BW. BW stands for bandwidth. Therefore, *h*[0], *h*[1],…, *h*[*d* − 1] can be used to replace response vector *h*. A large number of response vectors *h* constitute a response space *h*1, *h*2, *h*3,…. The most important part in the test generation algorithm is to find out the function of linear classification hyperplane. Therefore, here suppose that the expression of a linear classification hyperplane is as follows:

(5) $$\begin{array}{c} f\left(H\right)=\overset{d-1}{\underset{m=0}{\sum }}c_{m}\cdot h\left[m\right]-c_{d}=0. \end{array}$$

In Formula (5), *c* = (*c*_*d*−1_, *c*_*d*−2_,…, *c*_0_) and *c*_*d*_ can be obtained from the classification algorithm, respectively. Here, *c* is used to replace stimulation vector *x*. Then, Formula (5) can be rewritten into

(6) $$\begin{array}{c} f\left(H\right)=y\left[d-1\right]-c_{d}=0. \end{array}$$

Therefore, the coefficient of linear classification hyperplane *c* = (*c*_*d*−1_, *c*_*d*−2_,…, *c*_0_) is taken as test sequence. *c*_*i*_ represents different voltage and *c*_*d*_ is taken as threshold value. Thus, whether the circuit is fault can be judged by comparison of these values.

The ELM algorithm is not a linear classification algorithm. Therefore, test signal *c* and threshold value *c*_*d*_ cannot be directly obtained through classifier training. To this end, the authors put forward a test signal and threshold value calculation method based on ELM.

We suppose that

(7) $$\begin{array}{c} t_{\text{ELM}}\left(h\right)=\beta \cdot h\left(x\right). \end{array}$$

If *c* is available to make

(8) $$\begin{array}{c} c\times h=t_{\text{ELM}}\left(h\right), \end{array}$$

then

(9) $$\begin{array}{c} c=t_{\text{ELM}}\left(h\right)\times h^{-1}. \end{array}$$

Consider *t*_ELM_(*h*) = *β* · *h*(*x*). Here, *h* stands for impulse response sampling vector. As *h* is response impulse (i.e., *h* is not zero), generalized inverse certainly exists, because *β* can be obtained through ELM training. At the same time, the following formula can be obtained according to Huang's definition:

(10) $$\begin{array}{c} h\left(x\right)={\left[\begin{array}{ccc} g\left(w_{1}\cdot x_{1}+b_{1}\right) & \cdots & g\left(w_{\tilde{N}}\cdot x_{1}+b_{\tilde{N}}\right)\\ \vdots & \cdots & \vdots \\ g\left(w_{1}\cdot x_{N}+b_{1}\right) & \cdots & g\left(w_{\tilde{N}}\cdot x_{N}+b_{\tilde{N}}\right) \end{array}\right]}_{N\times \tilde{N}}. \end{array}$$

According to the theorem in ELM, the learning process of *h*(*x*) is different from the previous neural network algorithm. Input weight *w*_*i*_ and implicit threshold value *b*_*i*_ in Formula (10) can be obtained by random distribution. Once learning starts, the learning process of *h*(*x*) is unnecessary for parameter adjustment. Moreover, all parameters remain unchanged. At the same time, activation function *g*(*x*) is known, and thus it becomes a given parameter after the completion of learning. Thus, the coefficient *c* of linear classification hyperplane can be worked out according to (9) and (10). Thus, test signals are obtained. At last, the following formula can be obtained by combining Formulas (3), (6), and (9):

(11) $$\begin{array}{c} c_{d}=0. \end{array}$$

Formulas (9) and (11) are mathematical expressions based on the ELM test generation algorithm in this paper.

Based on the abovementioned analysis, learning algorithm proposed for test signal generation has obviously less computational complexity. And, in [2], coefficients *c* and *c*_*d*_ will be calculated. Computational costs of this process mainly come from calculation of Lagrange multiplier *a*, while the calculating process is equivalent to solving matrix with a size of (2*N* + 1)×(2*N* + 1) × 2. Solution to linear equations with a size of $\tilde{N}\times \tilde{N}\;(\tilde{N}\ll N$) is the main cause of computational cost of algorithm proposed in this paper. Moreover, in algorithm proposed in this paper, only one group of coefficient *c* will be calculated.

### 3.5. Novel Test Structure

**Figure 3:**
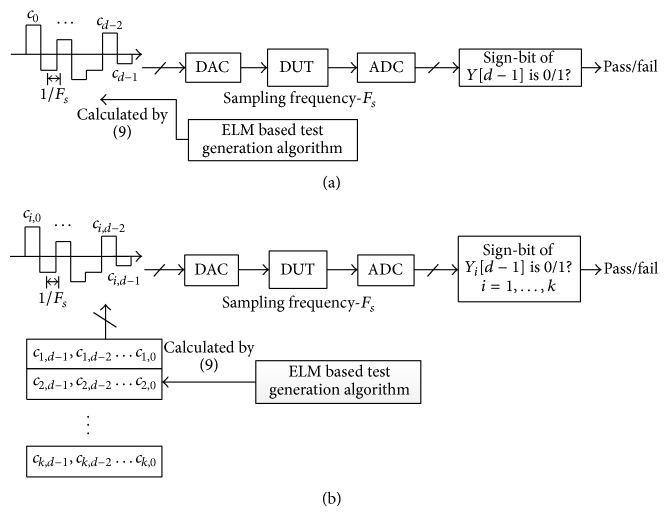
(a) Application of test sequence to DUT for classification. (b) Test structure of our proposed scheme. The entire test consists of *k* test sessions and subscript *i* = (1,…, *k*) denotes the *i*th session.

As Section 3.4 proposes a novel process of coefficient calculation, a novel test process is also proposed as shown in Figure 3.

In general, one hyperplane is used to tell between one kind of parameter fault circuit and normal circuit. Multiple hyperplanes are required for discrimination in order to satisfy different kinds of parameters fault. Therefore, the simplified case of using one hyperplane for discrimination, which corresponds to one kind of parameters, can be easily extended to a case of using multiple hyperplanes which correspond to different kinds of parameter faults. In Figure 3(a), test structure is shown by one test set. Test set *c*_*d*−1_, *c*_*d*−2_,…, *c*_0_ can be calculated with (9) and prestored in test equipment, and the output response *y*[*d* − 1] is at time instance *n* = *d* − 1 to the test set. As the output of ADC is based on signed binary arithmetic operation, the condition *y*[*d* − 1] > 0 (*c*_*d*_ = 0) can be modified into a condition in which sign-bit of *y*[*d* − 1] is 0. As shown in Figure 3(b), a novel test structure that uses multiple test sets and corresponds to different parameter faults is shown.

## 4. Structure and Steps of the Algorithm

**Figure 4:**
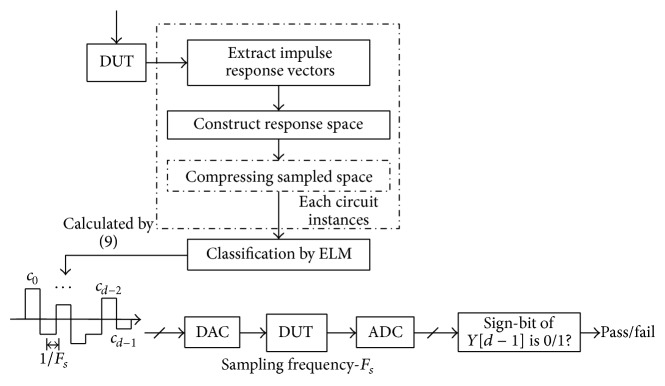
Single fault structural diagram of the test generation algorithm based on ELM.

**Figure 5:**
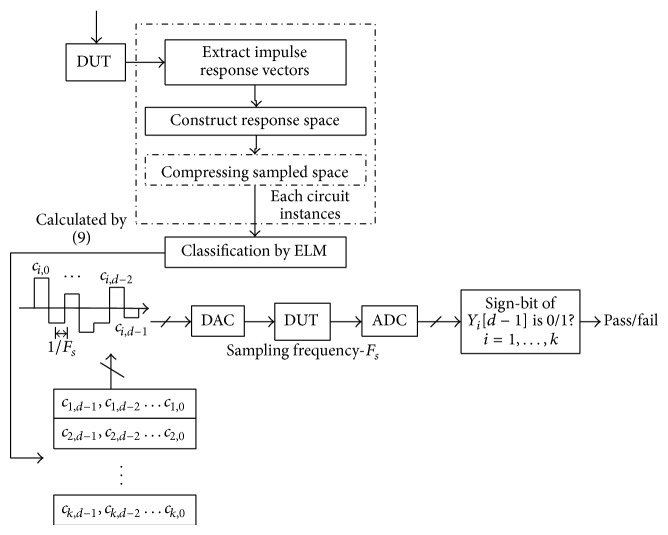
Multifault structural diagram of the test generation algorithm based on ELM.

Upon a summary of above paragraphs, various parts and structural diagrams of the test generation algorithm based on ELM proposed in this paper are shown in Figures 4 and 5. Its operational steps are given as follows.

**Step 1 .**

Sample CUT with sampling frequency *F*_*s*_ including normal state and various fault states of circuits and obtain abundant impulse response vectors and thus obtain impulse response spaces in different states; on the premise of guaranteed precision, impulse response spaces in different states can be subject to isometric compression.

**Step 2 .**

Introduce ELM classifier and perform classification training for impulse response spaces in various states, so as to obtain various known parameters required, namely *h*(*x*) and *β*; then, work out *c* according to Formula (9). Here, *c* = (*c*_*d*−1_, *c*_*d*−2_,…, *c*_0_) stands for test sequence.

**Step 3 .**

Use DAC to stimulate CUT with the test sequence obtained in Step 2; then, judge whether this state is satisfied and work out *c*_*d*_ = 0 according to Formula (11); it is only necessary for this algorithm to through a binary ADC with sign bit. Judge whether the sign bit of this output sequence is 0 or 1, namely, whether it meets the state of this circuit.

**Step 4 .**

Circuits always have multiple states, including normal state and multiple fault states. Therefore, there are always several values for *c* obtained from Step 2, as shown in Figure 5. To judge whether the circuit is normal or not, this needs to output several test sequences from tested circuits through DAC. Upon judgment for several times in Step 3, whether the circuit is normal or not can be judged. If it is abnormal, the type of fault state can be judged.

Circuit fault judgment via the analog circuit test generation algorithm always needs to operate Steps 2 and 3 for many times. Therefore, the efficiency of the whole algorithm will be directly influenced by the classifier's training test speed and parameter calculation speed as well as the parameter configuration speed of Step 3. According to the analysis in Section 3.3.1, the classification speed is relatively fast for ELM itself. According to the contents of Sections 3.4 and 3.5, there is only a need to work out the solution to *c* in the test generation algorithm in this paper. It is unnecessary to work out the solution to *c*_*d*_. Therefore, it is only necessary to calculate and configure a group of parameters in the process of signal generation and test. For the first two algorithms, however, two groups of parameters need to be calculated and configured. Therefore, certain improvement is made to the speed of the algorithm in this paper. In terms of precision, trade-off parameters of ELM have smaller influence on test precision against trade-off parameters of SVM during compression of sample space according to the analysis in Section 3.2. Therefore, the test generation algorithm based on ELM has some advantages in terms of precision during signal generation when compared with the algorithm based on SVM. Due to reduction of parameter calculations in the test generation process in terms of the algorithm in this paper, the deviation of calculation of parameters introduced into the test process from the classification process is decreased. As a result, the precision is improved to some extent. The performance of the algorithm will be verified in the experiment in the next section.

## 5. Examples

This section illustrates ideas of this paper by simulation. The entire simulation is completed on a personal computer with a 3-GHz processor and 2-GB RAM. Programs used in simulation are developed by authors in MATLAB 7.1 and OrCAD 10.5.

### 5.1. Example 1

**Figure 6:**
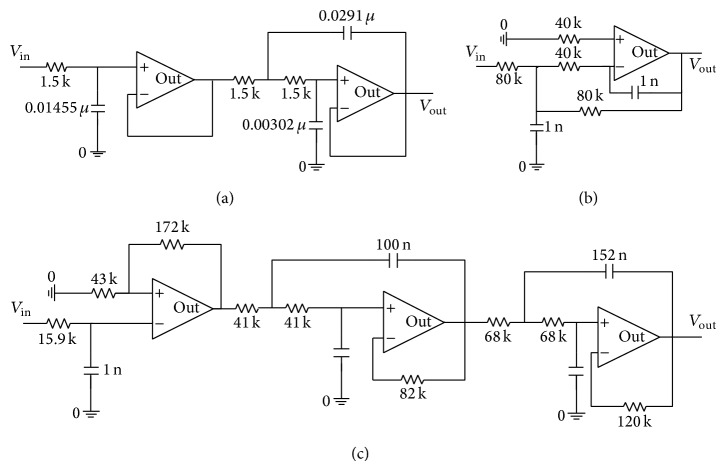
(a) A three-pole active filter. (b) A two-pole active filter. (c) A five-pole active filter.

**Figure 7:**
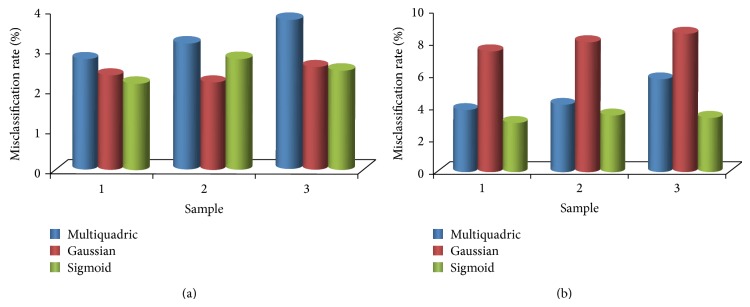
(a) Misclassification rates of ELM-based algorithm with different mapping functions for circuit in Figure 6(a), when the number of impulse-response samples is 30. (b) Misclassification rates of ELM-based algorithm with different mapping functions for circuit in Figure 6(a), when the number of impulse-response samples is 5.

ELM is one of the best classification algorithms based on activation functions. Without loss of generality, testing set misclassification of the test generation method is shown by circuits in Figure 6(a) corresponding to different mapping functions as shown in Figure 7(a). For a normal circuit, tolerance range of all parameters is [−10%, 10%]. For fault circuits, parameters of components are beyond the tolerance range [10%, 50%]∪[−50%, −10%]. Many sampled vectors can be acquired from circuit shown in Figure 6(a), and an impulse-response space is constructed. Each sampled vector is obtained by sampling the impulse response of a circuit instance. The amount of each sample is set to be 30, and impulse-response space can be divided into training or testing sets.

Each sampled vector of training sets is labelled as “passed” or “failed” according to circuit specifications. Testing set classification can be derived by setting the sign-bit of output response as zero. Furthermore, to determine effects of averagely compressed sample space, sampled vectors are established by averagely compressing previous impulse-response space, in which numbers of each sample and new impulse-response space are set to be 30 and 5, respectively. Here, trade-off parameters in different mapping functions are of default assignment.  Figure 7(b) shows the testing set misclassification of test generation method compared with different mapping functions.

Figure 7(a) shows rates of testing set misclassification of various mapping functions after applying ELM-based algorithm proposed in this paper to circuit in Figure 6(a). Misclassification rates include the ratio of the number of misclassified passed instances to the number of instances labelled as passed and the ratio of the number of misclassified failed instances to the number of instances labelled as failed instances. As shown in Figure 7(a), compared with ELM with multiquadric function, ELM with both Gaussian kernel function and Sigmoid activation function performs better in misclassification for the total population (%) which includes both passed instances and failed instances, when the number of impulse-response samples is 30. However, according to Figure 7(b), in the case of compressing (the number of impulse-response samples is 5), ELM with Sigmoid activation function has better misclassification than that with both Gaussian kernel function and multiquadric function. Therefore, Sigmoid activation function is a mapping function that suits ELM-based algorithm proposed in this paper.

### 5.2. Example 2

To compare with previous algorithms conveniently without loss of generality, three kinds of circuits are used in Figures 6(a), 6(b), and 6(c) to show the performance including misclassification rates and time cost, between SVM-based and ELM-based algorithms. Normal and fault circuit instances can be built by assigning parameters to components, and all normal parameters fall in their respective tolerance ranges. Parameter ranges of normal and fault circuits are assigned by the same way as in Example 1. The number of impulse-response samples is set to be 30, and simulation processes are implemented the same as in Example 1. Here, trade-off parameters in both ELM and SVM are assigned by default.

**Figure 8:**
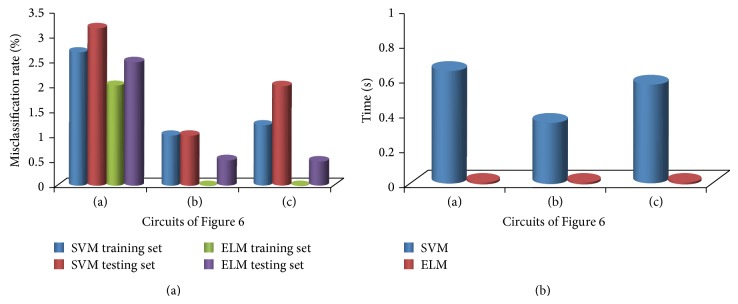
(a) Misclassification rates of SVM- and ELM-based algorithms for circuits in Figures 6(a), 6(b), and 6(c), when the number of impulse-response samples is 30. (b) Time cost of SVM- and ELM-based algorithms for circuits in Figures 6(a), 6(b), and 6(c), when the number of impulse-response samples is 30.

Figures 8(a) and 8(b) show time cost of circuits in Figures 6(a), 6(b), and 6(c) between SVM-based and ELM-based algorithms and misclassification rates including both passed and failed testing and training sets. Results show that ELM-based algorithm proposed in this paper as shown in Figure 8(a) can enhance the precision slightly, compared with SVM-based algorithm. Furthermore, as described in Section 3, the process of signal generator and test in ELM-based algorithm proposed in this paper are much simpler because of the computational complexity and classification theory of methods, and ELM-based algorithm can reduce time cost greatly as shown in Figure 8(b).

### 5.3. Example 3

In order to facilitate comparison with previous algorithms in the case of compressing sampled space, two kinds of circuits are used in Figures 6(b) and 6(c) to show misclassification rates between SVM-based and ELM-based algorithms with different numbers of impulse-response samples. Parameter ranges of normal and fault circuits are assigned by the same way as in Example 1, and simulation processes are also implemented the same as in Example 1.

The sampled vector can be written as (*h*_1_, *h*_2_,…, *h*_30_), as the number of impulse-response samples is set as 30 under no compressing. Numbers of impulse-response samples are set to be 15, 10, and 5 respectively, and new sampled vectors are constructed by extracting samples equidistantly from the impulse-response sampled space without compressing. Here, trade-off parameters in both ELM and SVM are of default assignment.

**Figure 9:**
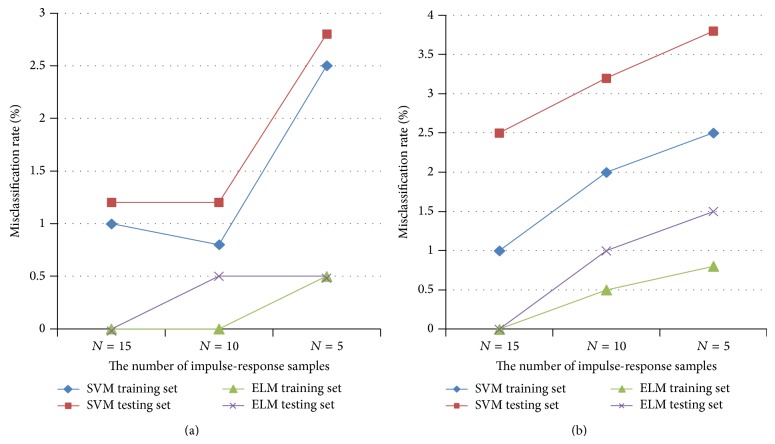
(a) Misclassification rates of SVM- and ELM-based algorithms for circuit in Figure 6(b), with different numbers of impulse-response samples. (b) Misclassification rates of SVM- and ELM-based algorithms for circuit in Figure 6(c), with different numbers of impulse-response samples.

Figures 9(a) and 9(b) show misclassification rates of both passed and failed testing and training sets for circuits as in Figures 6(b) and 6(c) between SVM-based and ELM-based algorithms. According to results, the ELM-based algorithm proposed in this paper can enhance the precision both for training and testing sets when the number of impulse response samples is reduced, because its trade-off parameters are less sensitive to classification accuracy than those of SVM-based algorithm in the case of compressing as described in Section 3.

### 5.4. Example 4

**Figure 10:**
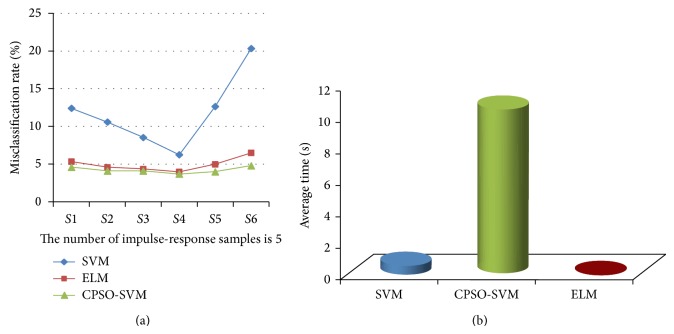
(a) Misclassification rates of different algorithms for circuit in Figure 6(a), when the number of impulse-response samples is 5. (b) Time cost of different algorithms for circuit in Figure 6(a), when the number of impulse-response samples is 5.

In the case of compressing sampled space, for circuit in Figure 6(a), misclassification rates increase the most as the number of impulse-response samples decreases to 5. So, circuit in Figure 6(a) is used to compare the misclassification in the case of compressing sampled space. With 5 impulse-response samples, we can construct 6 different sets of sampled vectors, namely, *S*1(*h*_1_, *h*_7_, *h*_13_, *h*_19_, *h*_25_), *S*2(*h*_2_, *h*_8_, *h*_14_, *h*_20_, *h*_26_), *S*3(*h*_3_, *h*_9_, *h*_15_, *h*_21_, *h*_27_), *S*4(*h*_4_, *h*_10_, *h*_16_, *h*_22_, *h*_28_), *S*5(*h*_5_, *h*_11_, *h*_17_, *h*_23_, *h*_29_), and *S*6(*h*_6_, *h*_12_, *h*_18_, *h*_24_, *h*_30_), respectively. For different sets of sampled vectors in the case of compressing, Figure 10(a) compares misclassification rates and time cost among ELM-based, SVM-based, and chaos particle swarm optimization- (CPSO-) SVM-based [24] algorithms, in which parameter optimization is applied to trade-off parameters of *C* and *r* in SVM. Here, trade-off parameters in both ELM and SVM are also of default assignment, and CPSO-SVM is a SVM algorithm based on parameters optimized to trade-off parameters.

The ELM-based, SVM-based, and CPSO-SVM-based algorithms are compared in Figures 10(a) and 10(b) in terms of misclassification rates and time cost for different sets of sampled vectors, in the case of compressing sampled space with default trade-off parameters. Results of Figure 10(a) show that ELM-based and CPSO-SVM-based algorithms with default trade-off parameters can better enhance the stability and misclassification rates than SVM-based algorithm. However, as shown in Figure 10(b), ELM-based algorithm proposed in this paper takes the least time among these three algorithms, as optimization of trade-off parameters would take about 10 seconds. In general, the ELM-based algorithm proposed in this paper can enhance precision and stability and reduce time cost with a decreased number of impulse-response samples.

## 6. Conclusions

This paper puts forward an advanced test generation algorithm for analog circuits. As described in Section 3, the proposed algorithm classifies response space based on ELM, and a novel process of test generation algorithm including test signal generator and test is proposed. Existing test generation algorithms cannot save time or stabilize the accuracy efficiently when the number of impulse-response samples decreases although groups can be separated efficiently. Due to computational complexity and classification theory of methods, ELM-based algorithm proposed in this paper has much simpler processes of signal generator and test and trade-off parameters not sensitive to classification accuracy in the case of compressing. This algorithm can not only avoid precision reduction under compressing, but also save time efficiently. Furthermore, this algorithm is confirmed to have good performance through experiments.
